# Building capacity in implementation science research training at the University of Nairobi

**DOI:** 10.1186/s13012-016-0395-5

**Published:** 2016-03-08

**Authors:** George O. Osanjo, Julius O. Oyugi, Isaac O. Kibwage, Walter O. Mwanda, Elizabeth N. Ngugi, Fredrick C. Otieno, Wycliffe Ndege, Mara Child, Carey Farquhar, Jeremy Penner, Zohray Talib, James N. Kiarie

**Affiliations:** 1 College of Health Sciences, University of Nairobi, Nairobi, Kenya; 2 Department of Global Health, University of Washington, Seattle, USA; 3 Department of Medicine, University of Washington, Seattle, USA; 4 School of Medicine, University of Maryland, Baltimore, USA; 5 George Washington University Medical Center, The George Washington University, 2121 K Street NW, Washington, DC, 20037 USA

**Keywords:** Implementation science, Training, Fellowship program

## Abstract

**Background:**

Health care systems in sub-Saharan Africa, and globally, grapple with the problem of closing the gap between evidence-based health interventions and actual practice in health service settings. It is essential for health care systems, especially in low-resource settings, to increase capacity to implement evidence-based practices, by training professionals in implementation science. With support from the Medical Education Partnership Initiative, the University of Nairobi has developed a training program to build local capacity for implementation science.

**Methods:**

This paper describes how the University of Nairobi leveraged resources from the Medical Education Partnership to develop an institutional program that provides training and mentoring in implementation science, builds relationships between researchers and implementers, and identifies local research priorities for implementation science.

**Results:**

The curriculum content includes core material in implementation science theory, methods, and experiences. The program adopts a team mentoring and supervision approach, in which fellows are matched with mentors at the University of Nairobi and partnering institutions: University of Washington, Seattle, and University of Maryland, Baltimore. A survey of program participants showed a high degree satisfaction with most aspects of the program, including the content, duration, and attachment sites. A key strength of the fellowship program is the partnership approach, which leverages innovative use of information technology to offer diverse perspectives, and a team model for mentorship and supervision.

**Conclusions:**

As health care systems and training institutions seek new approaches to increase capacity in implementation science, the University of Nairobi Implementation Science Fellowship program can be a model for health educators and administrators who wish to develop their program and curricula.

## Background

Health care systems worldwide have failed to bridge the implementation gap between medical evidence and current health care practice, resulting in inefficient use of resources and sub-optimal quality of care [1]. The need to narrow the implementation gap is particularly critical for sub-Saharan Africa, as the region bears nearly a quarter of the global disease burden while having only 1 and 3 % of world’s financial resources and health care workforce, respectively [2]. Whereas, an increasing number of evidence-based interventions and funding, such as the Presidential Emergency Plan for AIDS Relief (PEPFAR), are becoming available for priority diseases that affect sub-Saharan African countries: many sub-Saharan countries have weak health care systems and lack capacity in implementing effective interventions [3]. According to the World Health Organization (WHO), it is imperative to focus on closing the implementation gap (also referred to as the “know-do” gap) in order to strengthen health systems and to ultimately achieve equity in global health [4]. National and international health agencies, including the National Institutes of Health (NIH), have identified the translation of evidence-based findings into practice in diverse health care settings as an integral component of health programs [5, 6]. This context has catalyzed an interest and urgency to understand the process of successful implementation of evidence-based practices, and as such, implementation science has emerged as a relatively new discipline.

**Fig. 1:**
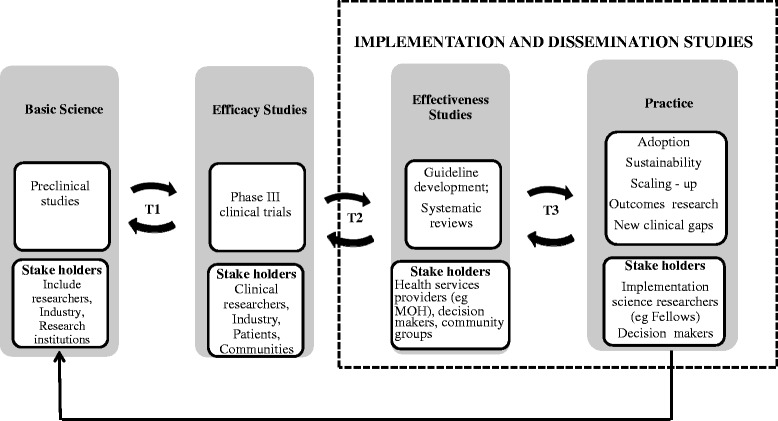
Phases of translation research cycle. T1—the first translational step characterized by discovery to a candidate with potential health application; T2—the second translational step applies mainly effectiveness studies and reviews for guideline development; T3—the third translational step applies strategies to move evidence-based interventions into practice^6,7^

Implementation science (IS) has been defined as the study of “the use of strategies to adopt and integrate evidence-based health interventions and change practice patterns within specific settings” [5]. As conceptualized in a model developed by the National Research Council and Institute of Medicine, implementation studies occupy a late phase of knowledge translation (Fig. 1) [7]. According to this perspective, implementation and dissemination studies are the final stages of the pathway from scientific discovery to practice. Implementation science occupies a distinct locus of knowledge translation, characterized by the channeling of scientific knowledge from basic discovery to testing for efficacy (sometimes termed translation 1), or from efficacy-tested interventions to testing for effectiveness (translation 2) to adoption in practice, sustainability, and moving to scale (translation 3) [8]. There is a glaring gap between empirical evidence and its application in health care programs in many countries including Kenya. Consequently, there is a compelling need for implementation science training to promote the uptake of proven interventions in African health care settings.

According to the NIH, progress in implementation science will require the training and mentorship of a new type of professional, advances in dissemination and diffusion of information on evidence-based interventions, strengthening linkages between scientists, program implementers, and policy makers, and agenda setting to identify local priorities [9]. Prior to our program, no sub-Saharan African countries, including Kenya, had a formal training program to build sustainable expertise in implementation science. For the University of Nairobi (UON) in Kenya, the only option for training in IS started recently through a relatively new Fellowship program that is run by a consortium of eight universities organized in four pairs, with the University of Nairobi paired with the University of Washington. The Afya Bora Consortium Fellowship relies on external funding, resources, and teaching to train a cohort of Kenyan students in IS over 12 months. The fellowship thus exists outside the structure of the UON.

Building local capacity to provide on-going training in IS requires a consistent pool of motivated trainees, clinical sites to conduct research, and teachers for both the theory and the practical application of IS. Training institutions in sub-Saharan Africa, such as UON, are challenged by a lack of faculty with relevant expertise, limited funding, and a lack of attachment sites staffed with experienced mentors for practical training. These barriers are compounded by the need for highly motivated trainees who can afford to take time away from their employment for graduate level training which typically lasts 1 to 2 years. Employers, stakeholders, and potential trainees are also deterred by a lack of awareness of career prospects in implementation science [10].

The objective of this article is to describe the approach used by the University of Nairobi to overcome these barriers and build local capacity for implementation science. This paper examines how UON leveraged resources from the Medical Education Partnership Initiative (MEPI), created an institutional program to provide training and mentoring in implementation science, built relationships between researchers and implementers, and identified local research priorities for implementation science. The goal of the training program is to improve the quality of health care in Kenya by producing implementation scientists with the capacity to design, implement, monitor, and evaluate evidence-based interventions in health care settings.

## Methods

### Ethical approvals

This paper describes an Implementation Science Fellowship program that delivers a novel curriculum aimed at producing researchers skilled in implementation science. As part of our evaluation plan for the program, a questionnaire was developed as a tool to assess both process and outcomes of the program. The questionnaire was administered on a voluntary basis to fellows, faculty, mentors, and administrators from the partnering institutions, all of whom were provided with full information on the evaluation, including the purpose and nature of the evaluation. Prior to obtaining verbal consent, the participants were assured that their personal information would be kept confidential, and that their identity would not be revealed. Fellows were further assured that there would be no negative consequences for not participating. Questionnaires were designed to take not more than 45 min to complete to minimize loss of time. The study was approved by the KNH/UON Ethical Review Committee.

### Establishing key partnerships to train in implementation science

Implementation science practice and training requires partnerships between researchers, health care providers, and policy makers, or more broadly between academia, health facilities, and government ministries. Establishing these partnerships served as the foundation for the program at the UON.

The University of Nairobi (UON) has well-established partnerships with the University of Washington (UW) and the University of Maryland, Baltimore (UMB). The partnership with the University of Washington began in the context of the AIDS International Training and Research Program (AIRTP), an initiative whose goal is to support training and HIV-related research. UON’s collaboration with UMB was launched as a component of the AIDS Relief Program, a consortium funded through the US President’s Emergency Plan for AIDS Relief (PEPFAR) to provide treatment services to HIV-infected individuals in Kenya and other African, Caribbean, and Latin American countries [11]. The three Universities leveraged their existing ties to forge the Partnership for Innovative Medical Education for Kenya (PRIME-K), a consortium funded by the Medical Education Partnership Initiative (MEPI). MEPI was a $150M investment of the US Government, to strengthen the health workforce in Africa. The University of Nairobi was one of 11 medical schools in sub-Saharan Africa to receive this substantial 5-year grant to build capacity in medical education, improve retention of health care workers in underserved areas, and build research capacity. The MEPI grant, which was launched in 2010, was part of President Obama’s Global Health Initiative in which country ownership was prioritized. As such, funding was provided directly to African medical schools enabling them to develop work plans based on their local needs and priorities. Building capacity in implementation science was identified at the outset as a key pillar for achieving the goals of PRIME-K.

**Fig. 2:**
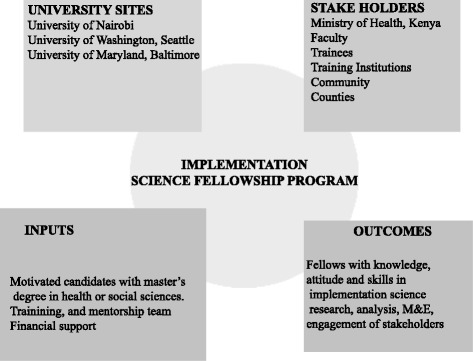
Structure of the Implementation Science Fellowship program

With funding in place, the leadership of the University then established key partnerships for the program. UON (University of Nairobi) trains its medical and health sciences students at the Kenyatta National Hospital (KNH). KNH is thus a natural partner for UON in most of its health-related initiatives. The mission of KNH “to optimize patient experience through innovative health care; facilitate training and research; and participate in national policy” articulates key tenets of implementation science. The hospital required little motivation to participate in the partnership. KNH is the setting for a large proportion of biomedical research in Kenya; however, few employees of KNH have led research. The prospect of strengthening the research capacity of KNH employees, through the IS training, was a major incentive for the hospital to participate.

Implementation science is ideally conducted in diverse settings to establish the feasibility of evidence-based approaches in different settings, including and especially places of routine care for the general population. As such, the PRIME-K leaders participated in a consultative process with the Kenyan Ministry of Health (MOH) to identify hospitals and health care settings across the country to participate in the program. The Ministry of Health employees at the selected sites were incentivized to participate by providing them appointments as adjunct faculty status with the UON. Adjunct faculty would thus work collaboratively with UON faculty on research projects in addition to training UON students during community-based clinical rotations. Partnerships to enable the IS training program thus formed between the University of Nairobi, KNH, the MOH, and northern schools (UMB and UW).

### Designing a locally sustainable and relevant program

Careful consideration to feasibility, sustainability, and local relevance was given during the design of the training program, which required aligning with national priorities and resources. In Kenya, the Ministry of Health has prioritized training in implementation science. In its policy document entitled “Kenya AIDS Strategic Framework (KASF),” the Kenyan Ministry of Health lists “priority intervention areas” which include the “increase [of] evidence-based planning and the use of implementation science outcomes to optimize programming and policy changes” [12]. Implementation science is accorded special emphasis in the preface to this national policy document [12]. Other priority areas identified in the KASF are social, behavioral, cultural, biomedical, scientific, and technological innovative interventions. This policy document is specific for HIV prevention, treatment, and impact mitigation. Priority setting was through a participatory process, initiated at the national level by the Ministry of Health, and involved diverse stakeholders including government agencies, international agencies, and civil society. Those who participated in the process included the Parliamentary Committee on Health, the National AIDS Control Council (NACC), the County governments represented by the Council of Governors Consultative Forum, faith-based organizations, population representatives (such as People Living with HIV and Persons with Disabilities), and civil society organizations. International agencies that provided technical and financial support to the process included the World Health Organization, the US Centers for Disease Control and Prevention (CDC), the United States Agency for International Development (USAID), and PEPFAR. UON was represented in the priority setting process by faculty who participate in the MOH Technical Working Group (TwG). NACC spearheaded the process, undertook an analysis of Kenya’s health care status and trajectory, and assessed the previous policy framework. A report was then submitted to the stakeholders for identification, deliberation, and ranking of priorities [12].

With funding and partners in place for investing in a locally relevant training program, the UON leadership sought to establish a program that could ultimately be sustained locally through graduates of the program. Various training models such as a Masters, Diploma, or Fellowship program were considered. Master programs typically require a lot of time on campus, making it difficult to maintain full-time work at the same time. Given that the target audiences for the program were those already with a Master’s degree, this approach also seemed unlikely to attract sustained interest. The Diploma option, on the other hand, was likely too short and may not allow adequate development of skills. The Fellowship approach, allowing students to remain in their place of work while completing their studies during evening hours, weekends, and online, seemed the best pathway to ensure a sustained interest from students and yet develop strong competencies in implementation science. The one substantive time commitment is a 3-month period where student in the early years of the fellowship (before in-house capacity is built) would go to one of the US Universities for didactic training.

The selection process was framed to achieve a balanced mix of fellows based on educational background, geographical place of work, and gender. Trainees were to be selected from UON, KNH, and MOH health facilities. The minimum qualification for entry was a Master’s degree in the health sciences including pharmacy, nursing, medicine, dentistry, and public health but not limited to health sciences. Applicants with a Master’s degree in biomedical sciences or social sciences were also eligible. Applicants were required to demonstrate motivation to pursue a career in implementation science, be able to attend training for 2 years if accepted, and provide a letter of support from their organization or institution. Since one of the program objectives was to expand the cohort of researchers at UON, KNH, and the MOH, preference was given to early-career applicants and those without established major research funding.

The curriculum for the program was based on an existing model from a partner school but contextualized for Kenya. Implementation science is a new discipline and is only taught at a handful of university departments globally [13]. As such curricula for implementation science are highly varied and depend on the training context [10, 14, 15]. There were no Kenyan or African universities offering training in implementation science prior to ours. One of our partners, UW, was an early adopter and developer of an implementation science curriculum, initially as a course unit within the Master of Public Health in Global Health program and subsequently in the PhD (Global Health) program. The implementation science curriculum at UW thus served as an initial model in developing the UON fellowship program. While some key competencies are similar to both the UW and UON implementation science curricula, the two programs differ substantially in a number of key design elements reflecting the different contexts. Differences in the two programs are found in the programs’ title, structure, duration, elements of the content, selection criteria for trainees, delivery channels, and management. For example, students in the UW’s program graduate with a PhD in Global Health, after 4–5 years of study including a 2-year course work in political methods, advanced political research, global program management, and evaluation which are not available in the UON IS fellowship program (Table 1).

**Table 1:** Comparison of curriculum requirements for UON and UW Implementation Science program

Curriculum requirement	UON	UW
Duration of program (years)	2	4–5
Certification awarded	Fellowship	PhD
Implementation Science Methods	Quantitative and qualitative methods taught	Advanced quantitative and qualitative methods taught
Political methods	Less emphasis, however skills in stakeholder analysis given	Advanced political research
Health program management and evaluation	Systematic reviews and comparative analyses of Kenyan health programs	Global program management and evaluation
Mentorship	Mentors from Kenya and USA	Mentors from USA
Implementation Science case studies	Mainly Kenyan cases such as voluntary medical male circumcision in a comparative global context	Broad spectrum of case studies from Americas, Asia, and Africa

Leadership for the UON implementation science curriculum development was entrusted to a coordinating committee. In designing the educational framework for the fellowship program, the coordinating committee extensively consulted with UON partners and stakeholders and applied a combination of educational principles to develop the curriculum. In particular, competency-based medical education (CBME) and adult-learning principles underpin the curriculum. Other curriculum elements that were added and emphasized include innovative teaching strategies, content related to the Kenyan health care system, inter-professional team training, assessment of learning needs, mentoring, development of research skills, and evaluation of the processes [16].

The core competencies of the program (Table 2) were aligned to the educational goals of training implementation scientists with the capacity to identify local research priorities for implementation science, to be able to design, implement, monitor, and evaluate evidence-based interventions in health care settings, and to partner with both researchers and implementers. Through a consultative process, the core competencies of the program were identified. To facilitate the preparation of the first curriculum draft for discussion, the coordinating committee initially solicited comments and materials regarding competencies, from UON faculty teaching IS in the Afya Bora Fellowship program and from faculty teaching IS at UW. A baseline assessment survey was conducted among UON faculty and graduate students to identify curriculum needs. The draft curriculum was shared with stakeholders for additional input, including partnering institutions, MOH, KNH, UON faculty, UON curriculum experts, and Afya Bora fellowship program graduates.

**Table 2:** Curriculum competencies for Implementation Science Fellowship program

Competency	Content	Mode of delivery
Implementation science (IS) knowledge, skills, and experience	Principles and development of the theoretical approaches and models of IS. Knowledge dissemination, translation, and diffusion research. Comparative effectiveness research. Comparative operations research.	Modular courses in knowledge translation, dissemination, and implementation. Workshop series. e-Seminar series. Attachment to partner institutions
IS research methods	Qualitative and quantitative research methods: paradigms, design, implementation, data analysis, writing.	Research project. Modular courses, Workshop series in writing proposals, grants, and manuscripts. Individual research project.
IS analysis and review	Systematic reviews, analyses, and comparison of complex IS interventions, pragmatic trials, health programs, health care policy, health care systems, and resource utilization	Modular courses, e-seminar series, workshops, team work
Developing, monitoring, evaluating, and sustaining IS Interventions	Design, measurement, evaluation, scale-up, spread, and sustainability of effective interventions. Research-to-policy gaps. Policy-to-programming, development of innovative approaches to improve health care delivery. Appropriate adaptation of context relevant interventions.	Modular courses. Workshop series. e-Seminar series. Individual research project. Attachment to partner institutions
Meaningful engagement of and collaboration with stakeholders	Communication, teamwork, collaboration, responsible conduct of research, and implementation.	Modular course, roles and role modeling, mentorship.

Comments and suggestions were synthesized and discussed at two interactive workshops, where faculty, stakeholders (including MOH), and educational experts (including the Dean of the UON School of Education) participated, to further deliberate and refine the educational goals, competencies, curriculum content, and teaching strategies for the IS program. The final curriculum aims for a mastery of each of the competencies and skills (Table 2) through multiple curriculum delivery modes that combine lectures, seminars, group work, practical exercises, themed workshops, team-based learning, mentoring, attachment at the partners’ sites, and individual research projects. To support distance learning and to engage distance experts from our partner institutions, we apply a variety of innovative web-based technologies to deliver content including webcasts, emails, online resources, and teleconferencing. Each fellow is provided with a laptop computer to enhance access to IT-dependent courses.

The curriculum content includes core materials in implementation science theory, methods, and experiences (Table 2) taught by faculty from one of the three academic institutions. Foundational topics are taught by UON faculty with additional webcast lectures from UW. Core topics taught by local faculty include the theoretical models and historical development of implementation science, knowledge translation, dissemination, diffusion, communication, adaptation, adoption, scalability, fidelity, and responsible conduct of research. Topics taught by faculty from UW include global health, policy development and advocacy, program management and leadership, social marketing, dissemination research, impact evaluation, operations research, economic analysis, and systematic reviews of IS interventions. UW also provides case studies of specific programs such as polio eradication, malaria control, male circumcision for HIV prevention, and safe motherhood. UMB faculty provides training in qualitative and quantitative research with topics in research design, epidemiology, effectiveness research, data analysis, measurement, monitoring, and evaluation. The choice of the fellowship training model means the program can focus on IS topics, instead of a more general research training curriculum that would be part of a Master’s degree. Since the target audience was individuals with a graduate degree who would likely already have foundational training in research, a broad overview of research methods was considered sufficient for this program. The program therefore includes a mandatory workshop series on diverse research topics, including responsible conduct of research, grant writing, proposal writing, mentorship, manuscript writing, and publishing.

For individual research projects, fellows identify research topics in implementation science that are relevant to the local context or to the host organization of the fellow such as the MOH health care facilities. Fellows then develop proposals and undertake data collection, analysis, and report writing with guidance from mentors and supervisors. The program has adopted a team mentoring and supervision approach, in which a fellow is matched with a local mentor, a local research supervisor, and an additional mentor or supervisor from the partner institutions (either UW or UMB). In certain cases, the mentor may also be the research supervisor. A key innovation in our program is organization of “mentoring workshops,” at which the fellows, the local mentors, and supervisors get further opportunities to interact and establish and assess progress towards the objectives.

## Results and discussion

The first cohort of fellows admitted to the program includes six individuals from diverse backgrounds including public health (2), pharmacy (2), medicine (1), and nursing (1). One successful applicant was unable to join the program for personal reasons. Of the five participants, two were female. The second cohort has five trainees, with professional qualifications in public health (1), pharmacy (2), dental sciences (1), and nursing (1) (Table 3)

**Table 3:** Characteristics of the trainees admitted into the Implementation Science Fellowship program

Characteristic	Cohort 1 (*n* = 5)	Cohort 2 (*n* = 5)
Educational and professional background		
Public health	1	1
Nursing	1	1
Medicine	1	0
Dental sciences	0	1
Pharmacy	2	2
Geographic place of work		
Urban (Nairobi)	5	3
Rural (outside Nairobi)	0	2
Gender		
Male	3	1
Female	2	4

.

The first cohort of fellows has developed projects in the areas of both dissemination and implementation. The research topics include (i) determinants of contribution of research in Kenyan universities in informing National HIV policies and practice, (ii) evaluating and improving the quality of obstetric and neonatal care at Kenyan urban and rural health care facilities, (iii) sustainability and cost-effectiveness of antimalarial medicines program in a Kenyan county, (iv) correlates of organizational cultural factors and clinicians’ practice with low early mortality of patients initiated on ART in Kenya, and (v) fidelity of anticoagulants prescription practice to the guidelines.

The training program incorporates a monitoring and evaluation framework to measure progress towards the attainment of the program goals. The program administered a questionnaire to fellows in years 1 and 2 to solicit feedback and to assess various aspects of the training program including perceived value and progress towards desired objectives. Analysis of survey results revealed a high degree of fellows’ satisfaction with most aspects of the program, and most fellows indicated that they would recommend the program to their colleagues (Table 4). Most fellows expressed high satisfaction with the mentorship program and would prefer the existing mentorship arrangement to be extended. Respondents also approved the duration of the program, the attachment sites, and the scheduling arrangements of the course. Some respondents indicated that they were already applying the skills gained during the course at their home institutions. One respondent wrote: “*Because of this training, I have been selected by my organization to participate in reviews of micro-research project proposals at my work place*.”

**Table 4:** Ratings by fellows of various aspects of the implementation science program

	Mean	SD	Range
Program logistics and administration^a^			
Adequacy of information provided before the course	4.24	0.51	3–5
Time table and scheduling arrangement for the course	3.97	0.73	3–5
Duration of the training program	4.81	0.47	3–5
Satisfaction with the attachment site	4.97	0.38	3–5
Attainment of program objectives and mentorship^b^			
Likelihood of extending the existing mentorship arrangement	4.86	0.36	3–5
Likelihood of recommending the mentorship program to colleagues	4.93	0.58	3–5
Self reported individual achievement of program objectives	3.96	0.62	3–5

^a^1 = very dissatisfied; 2 = fairly dissatisfied; 3 = 50/50; 4 = fairly satisfied; 5 = very satisfied

^b^1 = very poor; 2 = poor; 3 = fair; 4 = good; 5 = very good

### Post-fellowship activities and career development

The career development opportunities for fellows who have completed the program are promising. Four fellows have embarked on research careers by enrolling in PhD programs in dissemination and implementation science research, three fellows have secured promotions, and one became a Departmental Chairperson. Three fellows have secured further funding to enable them continue with research.

Most (85 %) fellows identified implementation science research as a component of their work activity. Fellows have disseminated their research to general and specialist audiences at international conferences and published their work in peer-reviewed journals. One fellow regularly contributes a newspaper column with implementation science themes to a general audience. Fellows also contribute to broader research activities such as through membership in departmental research committees and adjunct professorships in partner institutions.

### Impact on the University of Nairobi

Representatives of the UON describe the fellowship program as a faculty recruitment and retention strategy by providing opportunities for growth and recognition. All of the fellowship recipients are currently still at UON (on the other hand, one applicant who was not awarded has gone into private practice). One fellow changed employment from a Ministry of Health hospital to a university teaching hospital, and another contributes as faculty in the Afya Bora Fellowship program. A key finding is that the fellows are regarded by representatives of UON as increasing the knowledge base and capacity of the UON to conduct research in an emerging field and enhancing institutional recognition through academic output.

### Expanding networks

Fellows and institutional representatives reported that the training program expanded their network. Most (90 %) fellows reported retaining contact with mentors and supervisors in the partnering institutions. Recently, UON implementation science researchers built on the program and the linkages with partnering institutions and MOH to win a new NIH grant in implementation science. UON has a growing community of implementation science researchers in diverse fields including mental health, child health, nursing, pharmacy, and dental sciences.

### Challenges and enablers

The strengths of the fellowship program include leveraging existing partnerships with the University of Washington (UW) and the University of Maryland, Baltimore (UMB) (Fig. 2). The collaborative training approach offers diverse experiences, perspectives, and linkages to the trainees. The training model further offers opportunities for international collaboration among institutions, professionals, and disciplines—key elements for enhancing global health. The challenge of international partnerships is the distance between fellows and mentors residing in different geographic locations which can complicate and hinder communication. We have addressed this challenge by the innovative use of information technology to enhance interaction between fellows and their mentors, as well as for teaching.

Sustainable sources of funding are another challenge for the program. The fellowship program was funded by the Medical Education Initiative (MEPI), an initiative of the President’s Emergency Plans for AIDS Relief (PEPFAR), also supported by the US National Institutes of Health (NIH). This time-bound source of funding covers tuition and students’ stipend. Fortunately, sources of funding for Kenyan implementation science researchers are increasingly becoming available. Funding is available from local agencies, such as the Consortium for National Health Research and the Ministry of Health [17]. There are also international agencies that encourage and support dissemination and implementation science research in Kenya and Africa including the NIH, the Special Programme for Research and Training in Tropical Diseases (TDR), and the United Kingdom’s Wellcome Trust [18–20]. Based on the positive experience of the first two cohorts and the anticipated success of their research efforts, the UON is considering converting the program into a postgraduate fee-paying program to sustain the program, while also seeking other partnerships to sponsor future fellows.

## Conclusions

The persistent gap between evidence-based health interventions and actual health care delivery particularly in low-resource settings underscores the critical need to develop a workforce skilled in implementation science. Such training will serve to strengthen the capacity for health care systems to move evidence-based interventions into diverse health care and community settings, improving both the efficiency and the effectiveness of health services.

In this article, we have described an Implementation Science Fellowship program that delivers a novel curriculum aimed at producing researchers in implementation science. The fellowship program applies a partnership approach that offers diverse experiences and perspectives and innovatively leverages information technology and a unique mentorship approach. Early findings suggest that this program has had a positive impact both on the participants and the host institution and could thus serve as a model to stimulate further expansion of this nascent field, especially in low-resource settings.
